# Effects of SSRI treatment on GABA and glutamate levels in an associative relearning paradigm

**DOI:** 10.1016/j.neuroimage.2021.117913

**Published:** 2021-02-28

**Authors:** B. Spurny, T. Vanicek, R. Seiger, M.B. Reed, M. Klöbl, V. Ritter, J. Unterholzner, G.M. Godbersen, L.R. Silberbauer, D. Pacher, S. Klug, M.E. Konadu, G. Gryglewski, S. Trattnig, W. Bogner, R. Lanzenberger

**Affiliations:** a Department of Psychiatry and Psychotherapy, Medical University of Vienna, Austria; b Department of Biomedical Imaging and Image-guided Therapy, High Field MR Centre, Medical University of Vienna, Austria; c Christian Doppler Laboratory for Clinical Molecular MR Imaging, Vienna, Austria

**Keywords:** GABA, Glutamate, MRSI, SSRI, Learning, Hippocampus

## Abstract

Impaired cognitive flexibility represents a widespread symptom in psychiatric disorders, including major depressive disorder (MDD), a disease, characterized by an imbalance of neuro-transmitter concentrations. While memory formation is mostly associated with glutamate, also *gamma*-Aminobutyric acid (GABA) and serotonin show attributions in a complex interplay between neurotransmitter systems. Treatment with selective serotonin reuptake inhibitors (SSRIs) does not solely affect the serotonergic system but shows downstream effects on GABA- and glutamatergic neurotransmission, potentially helping to restore cognitive function via neuroplastic effects. Hence, this study aims to elaborate the effects of associative relearning and SSRI treatment on GABAergic and glutamatergic function within and between five brain regions using magnetic resonance spectroscopy imaging (MRSI).

In this study, healthy subjects were randomized into four groups which underwent three weeks of an associative relearning paradigm, with or without emotional connotation, under SSRI (10mg escitalopram) or placebo administration. MRSI measurements, using a spiral-encoded, 3D-GABA-edited MEGA-LASER sequence at 3T, were performed on the first and last day of relearning. Mean GABA+/tCr (GABA+ = GABA + macromolecules; tCr = total creatine) and Glx/tCr (Glx = glutamate + glutamine) ratios were quantified in a ROI-based approach for the hippocampus, insula, putamen, pallidum and thalamus, using LCModel. A total of 66 subjects ((37 female, mean age ± SD = 25.4±4.7) for Glx/tCr and 58 subjects (32 female, mean age ± SD = 25.1±4.7) for GABA+/tCr were included in the final analysis.

A significant measurement by region and treatment (SSRI vs placebo) interaction on Glx/tCr ratios was found (p_cor_=0.017), with post hoc tests confirming differential effects on hippocampus and thalamus (p_cor_=0.046). Moreover, treatment by time comparison, for each ROI independently, showed a reduction of hippocampal Glx/tCr ratios after SSRI treatment (p_uncor_=0.033). No significant treatment effects on GABA+/tCr ratios or effects of relearning condition on any neurotransmitter ratio could be found.

Here, we showed a significant SSRI- and relearning-driven interaction effect of hippocampal and thalamic Glx/tCr levels, suggesting differential behavior based on different serotonin transporter and receptor densities. Moreover, an indication for Glx/tCr adaptions in the hippocampus after three weeks of SSRI treatment could be revealed. Our findings are in line with animal studies reporting glutamate adaptions in the hippocampus following chronic SSRI intake. Due to the complex interplay of serotonin and hippocampal function, involving multiple serotonin receptor subtypes on glutamatergic cells and GABAergic interneurons, the interpretation of underlying neurobiological actions remains challenging.

## 1 Introduction

A variety of neurological and psychiatric pathologies including major depressive disorder (MDD) are associated with an imbalance of neurotransmitter concentrations in the human brain. While MDD is mostly associated with serotonin disturbances, *gamma*-Aminobutyric acid (GABA) and glutamate, the main inhibitory and excitatory neurotransmitters in the brain, are also affected (Krystal, 2002; Sanacora, 2012). Several studies using magnetic resonance spectroscopy (MRS), a tool for the non-invasive in vivo quantification of biomolecules, showed altered neurotransmitter distributions in patients suffering from MDD compared to healthy controls. Reduced levels of GABA and glutamate were reported in several brain regions including the anterior cingulate cortex (ACC) (Auer, 2000; Pfleiderer, 2003), occipital cortex (Sanacora, 2002; Sanacora, 2004), prefrontal regions (Yildiz-Yesiloglu and Ankerst, 2006; Hasler, 2007) and anterior temporal structures (Block, 2009; Lener, 2017).

Deficits in several cognitive domains, including attention, short-term and working memory, cognitive flexability or processing speed (Porter, 2007; Hammar and Ardal, 2009; Lam, 2014; Zuckerman, 2018), are highly prevalent in MDD and are associated to altered neurotransmitter levels. Intact neural plasticity, the brain’s ability of functional and structural adaptions, is the cellular and molecular basis for cognitive function and memory formation, which can be affected by impaired neurotransmitter homeostasis. Memory formation is mainly driven by long-term depression (LTD) and potentiation (LTP) via glutamatergic synapses (Bliss and Collingridge, 1993 Bear and Abraham, 1996). Disturbed neurobiological adaptions of existing synaptic formations impedes cognitive flexibility in a process called relearning, as primarily reported in animal studies (Berman and Dudai, 2001; Bahar, 2003; Luchkina and Bolshakov, 2019). Hence, intact homeostatic plasticity (Grewe, 2017; Mendez, 2018) tends to play a crucial role and disruptions in both the GABAergic and glutamatergic system have an impact on relearning capacities (Villarejo-Rodriguez, 2010; Jia, 2016). Previous MRS studies found associations of memory performance and learning induced changes in a combined measure of glutamate and glutamine (Glx) (Hutcheson, 2012; Stanley, 2017; Lacreuse, 2018; Thielen, 2018) or a relationship between brain Glx levels and cognitive decline (Nikolova, 2017). Also, GABA levels have a crucial influence on learning and memory formation. Relationships of GABAer-gic neurotransmission and learning performance in motor learning (Blicher, 2015; Kolasinski, 2019), tactile discrimination (Puts, 2011) and associative learning (Spurny, 2020) were reported. Previously, we examined the effects of associative learning on brain GABA+/tCr (a combination of GABA and macromolecules in relation to total creatine) and Glx/tCr levels in a region-of-interest (ROI)-based approach using MR spectroscopy imaging (MRSI). No learning-induced changes in GABA+/tCr and Glx/tCr levels in the hippocampus, insula and thalamus could be found using a facial associative learning paradigm. However, a correlation of hippocampal GABA+/tCr ratios and retrieval performance could be shown (Spurny, 2020). The influence of GABAergic neurotransmission may be explained by interneural network regulation of memory acquisition and adult GABA-dependent neurogenesis in the hippocampus (Sibbe and Kulik, 2017; McKenzie, 2018) or so-called GABAer-gic interneural neuroplasticity (Kullmann and Lamsa, 2007; Lamsa and Lau, 2019; Topolnik and Camire, 2019).

A recovery of cognitive dysfunction in patients suffering from MDD was reported after a treatment with selective serotonin reuptake inhibitors (SSRIs), the first line pharmacological treatment option (Herrera-Guzman, 2010; McIntyre, 2013), that affects neuroplastic processes (Hayley and Litteljohn, 2013). While SSRI treatment leads to increased extracellular levels of serotonin, downstream effects on GABAergic and glutamatergic neurons were discovered (Skolnick, 1996; Sanacora, 2002; Bhagwagar, 2004; Riga, 2016). The tight coupling of serotonin, GABA and glutamate was extensively investigated in the hippocampus, since hippocampal glutamatergic and GABAergic neurons express various serotonin receptor subtypes (Dale, 2016). Moreover, it was demonstrated that serotonin modulates learning abilities in animals and humans (Chamberlain, 2006; Ogren, 2008). The involvement of serotonergic receptor cross-talk could be primarily investigated in the regulation and acquisition of emotional memories in mice (Ogren, 2008; Eriksson, 2012; Slipczuk, 2013). While functional magnetic resonance imaging (fMRI) studies of patients with depression have consistently shown overactivity in the frontolimbic circuitry, including the dorsolateral prefrontal cortex (dPFC) and hippocampus during working memory performance (Harvey, 2005; Walsh, 2007), SSRIs are thought to reduce glutamatergic overactivity in the hippocampus and facilitate dendritic spine formation via brain derived neurotrophic factor (BDNF) (Nibuya, 1996; Dincheva, 2012), calcium/calmodulin dependent kinase II (Elgersma, 2004) or changes in gene expression (du Jardin, 2016). Nevertheless, ambiguous results in human and animal studies were reported over the last decades: In humans, vortiox-etine, a serotonin modulator, has been demonstrated to improve processing speed, attention, executive function, learning and memory, as well as hippocampus-dependent memory measures (McIntyre, 2014 ; Mahableshwarkar, 2016). Moreover, the SSRI escitalopram was found to facilitate behavioral flexibility, reversal learning and to improve memory consolidation in healthy volunteers (Harmer, 2002; Brown, 2012). However, impaired hippocampus dependent generalization of past learning to novel contexts in depressed patients after paroxetine treatment (Herzallah, 2013) was reported. Furthermore, several animal studies showed inhibition of hippocampal LTP and adaptions of the NMDA receptor (Skolnick, 1996) after acute or chronic SSRI treatment and after direct application of serotonin (Malenka and Nicoll, 1999) as well as inhibiting effects on pyramidal neurons and increased local GABA transmission (Ropert and Guy, 1991; Turner, 2004).

Nonetheless, the interplay of serotonin, GABA and glutamate and their influence on memory formation in vivo remains elusive. Targeting this question, most MRS studies performed so far have focused on a few brain regions using single voxel spectroscopy in rather small cohorts. Hence, this study aims to investigate neuroplastic effects of relearning and SSRI treatment on GABAergic and glutamatergic neurotransmission within and between five brains regions in a double-blind placebo controlled study. To investigate the effects of associative relearning, with or without emotional connotation, on neurotransmitter levels based on our previous work (Spurny, 2020), participants underwent two different associative relearning paradigms under SSRI or placebo treatment. A MRSI sequence was used to examine neurotransmitter ratios in a ROI-based approach. Beside the hippocampus, the key player in memory formation, insula and thalamus, areas that are involved in memory processing of emotionally relevant input (Bliss and Collingridge, 1993; Van der Werf, 2003; Houtchens, 2007; Piras, 2010; Wang, 2014; Alkozei and Killgore, 2015; Quarto, 2016; Koenig, 2019), as well as, the putamen and pallidum, generally involved in reversal learning (Cools, 2002), MDD and known to exhibit neurotransmitter alterations, after SSRI treatment (Anand, 2005 Veer, 2010; Tao, 2013; Meng, 2014; James, 2017) were included in this analysis.

## 2 Methods

### 2.1 Study design

**Fig. 1:**
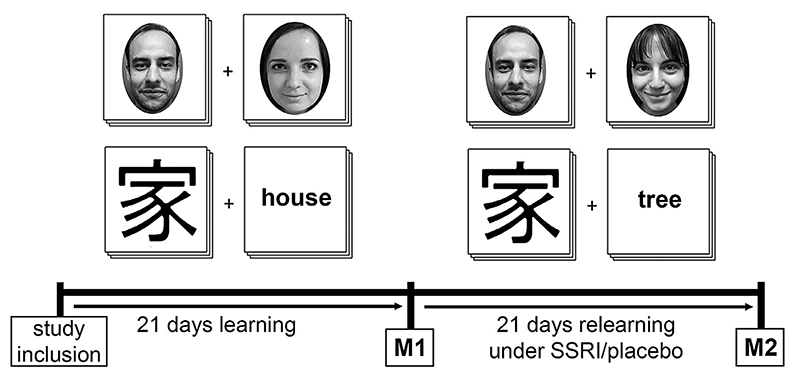
Study design. Participants completed a phase of 21 days learning either facial pairs or Chinese character associations prior to M1. Starting immediately after M1, study subjects underwent an associative relearning paradigm memorizing novel associations of previous content under SSRI or placebo treatment. SSRI = selective serotonin reuptake inhibitor, M1 = measurement 1, M2 = measurement 2.

79 healthy participants completed both MRSI measurements in this randomized double-blind, placebo-controlled longitudinal study. Subjects were divided into four groups which performed one of two different associative relearning paradigms for 21 days while receiving either SSRI or placebo treatment; group 1: character associative relearning + placebo ( *n*=20), group 2: character associative relearning + SSRI (*n*=20), group 3: facial associative relearning + placebo (*n*=21), group 4: facial associative relearning + SSRI (*n*=18). During the facial associative relearning paradigm participants had to memorize pairs of individual faces, while during the Chinese character associative relearning paradigm participants had to memorize random semantic associations between Chinese characters and German nouns (no translations). Prior to this stage of the study, participants had already completed 21 days of associative learning without medication (Spurny, 2020) (see Fig. 1 for details). Hence, novel associations of previously studied content were learned in the course of this stage under SSRI or placebo treatment. MRSI measurements were conducted on the first day (M1) and last (M2) day of relearning, with Ml before the first and M2 after the last online relearning session. GABA+ and glutamate concentrations were calculated in relation to total creatine (GABA+/tCr and Glx/tCr) (Mikkelsen, 2017), for five different regions (hippocampus, insula, putamen, pallidum and thalamus) to investigate treatment and relearning effects on metabolite ratios.

### 2.2 Subjects

A total of 66 participants (37 female, mean age ± SD = 25.4±4.7) could be included in the final analyses of Glx/tCr ratios while a total of 58 subjects (32 female, mean age ± SD = 25.1±4.7) were included in the analyses for GABA+/tCr ratios respectively. The rest of the data did not pass quality control. General health was assessed based on medical history, physical examination, electrocardiogram and routine laboratory parameters. The Structured Clinical Interview for DSM-IV Axis-I Disorders (SCID I) was administered by a psychiatrist in order to exclude any previous or current psychiatric diagnoses. Volunteers were free from internal, neurological or psychiatric disorders. Exclusion criteria included current or former substance abuse, lifetime use of SSRIs or related psychotropic agents, smoking, first-degree relatives with a history of psychiatric illness or substance abuse, color blindness, Mandarin, Japanese or related language skills, non-Caucasian ancestry or any contraindications for MRI. Urine drug and pregnancy tests were performed at screening and before each MRI session. This study was approved by the ethics committee of the Medical University of Vienna and was performed in accordance with the Declaration of Helsinki (1964). Participants gave written consent to this study and received financial reimbursement for their participation.

### 2.3 Associative relearning paradigm

Every participant had to complete a daily online associative learning paradigm over 21 days immediately before and during the treatment phase, resulting in a total of 42 days of learning. 39 participants were asked to complete a facial associative relearning paradigm. Facial images were taken from the “10k Adult Faces Database” (Bainbridge, 2013). In contrast to non-face objects, face perception and their expressing emotions as well as the evaluation of sympathy of faces presented, leads to an emotional connotation during the facial relearning paradigm (Tsao and Livingstone, 2008). Another 40 participants completed a Chinese character associative relearning paradigm, thus, without emotional connotation, see Fig. 1. However, during the treatment phase (SSRI vs placebo), the previous associations were extinguished and new associations were learned (relearning). A total set of 200 pairs was relearned under treatment. On each day, a different subsample of 52 pairs from the total set was presented. Pairs contained in each daily subset were predefined, ensuring that each pair was shown more than once (minimum: 2, maximum: 7, median: 6) over the course of 21 days, while their sequence within the daily set was randomly assigned. Single pairs were displayed for 5 seconds each during relearning sessions. A retrieval phase followed immediately after each relearning session, in which a matching image out of four options for a subset of 52 pairs of all previously learned associations had to be found. All possible answers were images of the total set of 200 pairs similar to (Spurny, 2020). Total relearning and retrieval time were approximately 15 min per day.

### 2.4 Escitalopram treatment and assessment of plasma levels

Subjects in group 2 and 4 received escitalopram (Cipralex® Lund-beck A/S, provided by the pharmacy of the Medical University of Vienna) 10 mg orally per day for 21 days. The other half of the study subjects (study groups 1 and 3) received placebo tablets for 21 days to serve as control. Venous blood samples were drawn from the cubital vein to assess citalopram plasma levels at 3 time points: 7 days and 14 days after therapy start as well as before the second MRI (i.e., 21 days after therapy start). Citalopram plasma levels were assessed with liquid chromatography-tandem mass spectrometry (LC-MS/MS) at the Clinical Department of Laboratory Medicine of the Medical University of Vienna.

### 2.5 Magnetic resonance imaging

MRI measurements were performed using a 64-channel head coil on a 3 Tesla MR Scanner (MAGNETOM Prisma, Siemens Medical, Erlangen, Germany) installed at the High-field MR Center, Department of Biomedical Imaging and Image-guided Therapy, Medical University of Vienna.

Structural T1-weighted images were acquired during each measurement using a standard magnetization-prepared rapid gradient-echo (MPRAGE) sequence (TE = 1800 ms, TR = 2.37 ms, 208 slices, 288 × 288 matrix size, slice thickness 0.85 mm, in-plane resolution 1.15 X 1.15 mm) for accurate placement of the volume of interest (VOI) during MRSI and mask extraction utilizing automated region-of-interest (ROI)-based analysis.

**Fig. 2:**
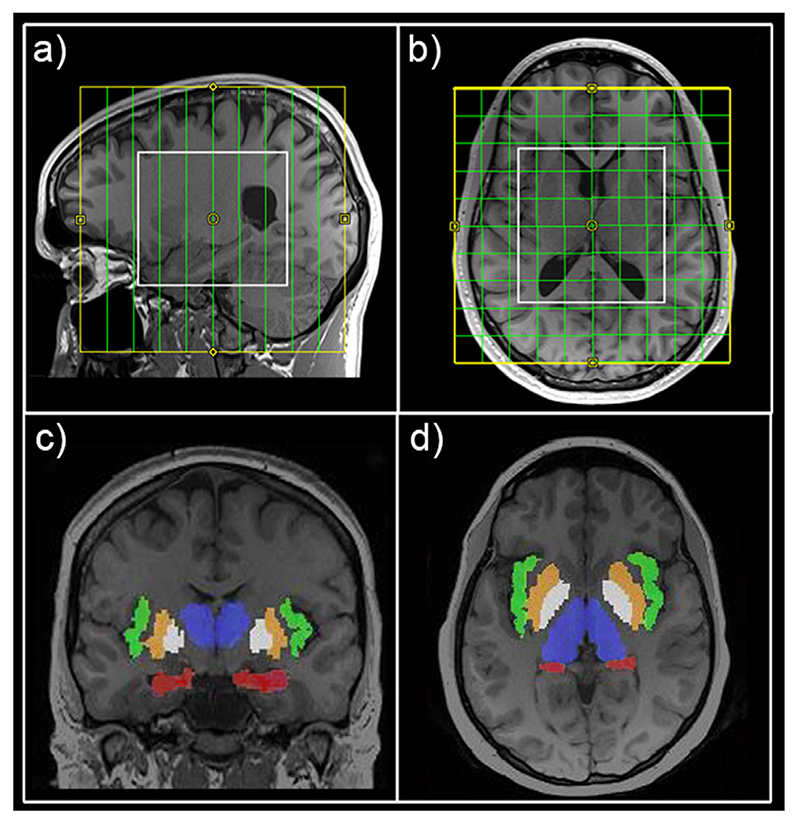
Magnetic resonance spectroscopy imaging position and exemplary mask extraction. Field of view (yellow) and volume of interest (white) are shown in sagittal (a) and horizontal (b) view. Exemplary automated mask extraction with FreeSurfer for the hippocampus (red), insula (green), putamen (orange), pallidum (white) and thalamus (blue) are shown in coronal (c) and horizontal (d) view.

For metabolic data acquisition, a spiral-encoded, 3D-MRSI sequence with MEGA-LASER (Mescher-Garwood-localization by adiabatic selective refocusing) editing, as described in Bogner et al. (Bogner, 2014a) was used. Real-time correction for rigid-body motion bias and correction of center frequency changes were applied (Bogner, 2014a; Bogner, 2014b; Heckova, 2018). The VOI was centered on the medial to posterior part of the corpus callosum and to cover regions of interest from the hippocampus to the insula bilaterally, with VOI = 80 (l-r) × 90 (a-p) × 80 (s-i) mm^3^ and field of view (FOV) = 160 × 160 × 160 mm^3^ (see Fig. 2). The acquired matrix size of 10 × 10 × 10 (i.e., approximately 4cm^3^ nominal voxel size) was interpolated to a 16 × 16 × 16 matrix (i.e., approximately 1 cm^3^ nominal voxel size) during spectral processing steps. Siemens advanced shimming procedure with manual adjustments was used. During the EDIT-ON acquisition, MEGA-editing pulses (60 Hz Gaussian pulses of 14.8 ms duration) were set to 1.9 ppm, editing the coupled 4CH_2_ triplet of GABA resonating at 3.02 ppm (Andronesi, 2010; Mullins, 2014; Bogner, 2017). VOI selection via LASER and low-power and wide-bandwidth GOIA pulses enabled MEGA editing with an echo time of 68 ms (Bogner, 2014a). For real-time correction, volumetric, dual-contrast, echo planar imaging-based navigators that update center frequency and head-position changes for each pair of EDIT-ON/OFF acquisitions were used (i.e. with a repetition time of 1.6 s, updated every 3.2 s). 32 acquisition-weighted averages and two-step phase cycling were employed for 3D-MRSI, resulting in a total scan time of 15:09 min.

### 2.6 Magnetic resonance spectroscopy data analysis

All spectra within the VOI were processed automatically with an in-house software tool using MATLAB (R2013a, MathWorks, Natick, MA, USA), Bash (4.2.25, Free Software Foundation, Boston, MA, USA), MINC (2.0, MINC Tools, McConnell Brain Imaging Center, Montreal, QC, Canada) and LCModel software (6.3-1, S. Provencher, LCModel, Oakville, ON, Canada). Two different simulated basis sets were created using the GAMMA library, one for the non-edited spectra (containing 21 brain metabolites, including total creatine (tCr)) and one for the difference spectrum (containing GABA+ and Glx among others) (Smith, 1994 Hnilicova, 2016), see supplementary figure 1. Cramer-Rao lower bounds (CRLB) thresholds were set at 30% and spectra were visually inspected. GABA+ and Glx concentrations were calculated as ratios to tCr (GABA+/tCr and Glx/tCr). Automated ROI-based analyses were performed as previously described (Spurny, 2019; Spurny, 2020). FreeSurfer (6.0, https://surfer.nmr.mgh.harvard.edu/) (Fischl, 2002 Desikan, 2006) was used for segmentation of structural T1-weighted images. In-house MATLAB codes were used to extract masks for each ROI (hippocampus, insula, putamen, pallidum and thalamus) per measurement. GABA+ and Glx (derived from the non-edited spectra) and tCr maps were interpolated to the resolution of structural images, overlaid with masks and mean GABA+/tCr and Glx/tCr ratios calculated for each ROI. ROIs with < 90% valid voxels, due to CRLB thresholds, were excluded from analyses for the particular subject.

### 2.7 Statistical analysis

SPSS Statistics (v26.0, 2010, SPSS, Inc., an IBM Company, Chicago, United States of America) was used for statistical analysis. Three-way repeated measures analyses of covariance (rmANCOVA) with neurotransmitter ratio (GABA+/tCr or Glx/tCr) at M1 and M2 as dependent variable, measurement (M1, M2) and ROI (hippocampus, insula, pallidum, putamen, thalamus) as within-subject factors and group (group 1: character assoc./placebo, group 2: character assoc./SSRI, group 3: facial assoc./placebo, group 4: facial assoc./SSRI) as between-subject factor, were performed to probe for effects of SSRI treatment and associative relearning, as well as possible interaction effects, on neurotransmitter ratios. The 4-level group factor was used to avoid an overly complex 4-way interaction in model building (relearning condition and substance as interacting factors). Gender and citalopram plasma levels at the time of M2 (mean-centered within the SSRI group) were included as covariates in the analyses. GABA+/tCr and Glx/tCr ratios were investigated in individual models. Bonferroni procedure was used to correct for multiple comparisons (2 models). In a subsequent analysis, the effects of treatment and relearning paradigm were tested separately. Hence, 3-way rmANCOVAs with measurement (M1, M2) and ROI (hippocampus, insula, pallidum, putamen, thalamus) as within-subject factors and SSRI/placebo treatment or relearning condition as between-subject factor, were performed to probe for effects of SSRI/placebo treatment or character/facial associative relearning, respectively, on each neurotransmitter ratio, independently. Again, Bonferroni procedure was used to correct for 2 comparisons since the additional models only constitute a re-parametrization of the above rmANCOVA with 4 group-levels. In case of significant treatment or relearning interaction effects, posthoc rmANCOVAs on the intra-subject differences of measurement and ROI with mean-centered citalopram plasma levels as covariate were performed to identify the responsible ROIs. Post-hoc tests were corrected using Sidak procedure (20 comparisons). Moreover, pair-wise 2-way rmANCOVAs of each ROI independently with measurement (M1, M2) as within-subject factor, SSRI/placebo treatment between-subject factor and mean-centered citalopram levels as covariate, were conducted on an exploratory basis to reveal effects of treatment neurotransmitter ratios (GABA+/tCr or Glx/tCr) at M1 and M2 in single ROIs. Because residuals were not consistently normally distributed for GABA+/tCr ratios (based on visual inspection of histograms and Kolmogorov-Smirnov test), rmANCOVA was repeated for GABA+/tCr ratios after rank transform (Conover and Iman, 1981), since log transform did not produce normally distributed residuals. Missing neurotransmitter ratio values, that failed to pass quality criteria (CRLB thresholds), were estimated by multiple imputation using ten repetitions. Mean imputed values were used in subsequent analyses (see Table 1 for number of imputations per ROI and neurotransmitter ratio).

## 3 Results

From a total of 79 participants, that completed both MRSI measurements, 3 participants in the SSRI treatment groups had to be excluded since the citalopram concentration was below the detection threshold at the day of the second measurement indicating irregular intake of the study drug. Moreover, subjects were excluded from the respective analysis, if the data quality, based on CRLB thresholds, of more than three ROIs was insufficient for one metabolite in one of the measurements, resulting in a total of 66 participants (37 female, mean age ± SD = 25.4±4.7) for the final analyses of Glx/tCr ratios and a total of 58 subjects (32 female, mean age ± SD = 25.1±4.7) for GABA+/tCr analysis. For the detailed number of subjects within each group see Table 2.

### 3.1 Associative relearning and treatment related neurotransmitter changes

Table 3 shows mean ± SD GABA+/tCr and Glx/tCr ratios for each ROI, measurement and group (1-4), respectively. RmANCOVA showed no significant interaction effect (measurement by ROI by intervention (treatment and relearning)) including all 4 groups for any neurotransmitter ratio (GABA+/tCr: p=0.349; Glx/tCr: p=0.118), but an effect of region (p_cor_ = 0.014) for Glx/tCr ratios. Since sphericity could not be assumed based on Mauchly’s test, Greenhouse-Geisser corrected results are given.

**Fig. 3:**
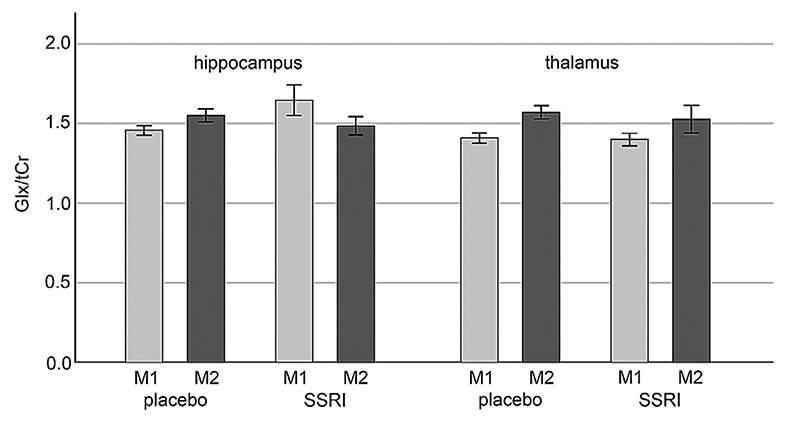
Hippocampal and thalamic Glx/tCr ratios. Mean Glx/tCr ratios in the hippocampus (left) and thalamus (right) for each measurement and treatment group (SSRI and placebo). Error bars representing the standard error. SSRI = combined SSRI treatment groups; placebo = combined placebo treatment groups; M1=measurement 1; M2=measurement 2.

Hence, in the subsequent analyses, groups were split by associative learning paradigms or treatment respectively (see Table 2 for detailed group size). Mean ± SD GABA+/tCr and Glx/tCr for each ROI and measurement for grouped cohorts (relearning and treatment groups) are shown in supplement Table 1. No significant associative relearning interaction effects (measurement by ROI by relearning condition) were found. Moreover, no effects of treatment could be reported for GABA+/tCr (p=0.284) ratios. However, a significant treatment (SSR/placebo) interaction (measurement by ROI by treatment) for Glx/tCr ratios (p_cor_ =0.017) could be revealed. Post-hoc rmANCOVAs revealed a significant hippocampus-thalamus interaction (p_cor_=0.046) and thus the differential behavior of these two regions as responsible for the effect. Pair-wise comparisons using rmANCOVA for each ROI individually showed significant reductions in hippocampal Glx/tCr ratios after SSRI treatment (p_uncor_=0.033). Mean hippocampal and thalamic Glx/tCr ratios for each measurement and treatment cohorts are displayed in Fig. 3.

## 4 Discussion

Here, we investigated the effects of associative relearning under SSRI or placebo treatment on brain GABA+/tCr and Glx/tCr ratios in young healthy subjects using MRSI in a double-blind longitudinal study design. We demonstrate a significant interaction of hippocampal and thalamic Glx/tCr levels after SSRI treatment showing differential effects on these ROIs. Moreover, reduced Glx/tCr ratios after SSRI treatment could be revealed in the hippocampus, when effects of treatment were probed independently in single ROIs, while changes in the thalamus and other ROIs investigated did not reach statistical significance. Similar to our previous work we could neither show changes of brain GABA+/tCr in any investigated ROI, after associative relearning or treatment (Spurny, 2020), nor effects of relearning on Glx/tCr ratios depending on relearning condition. Although, associative learning and relearning share many underlying neurochemical mechanisms, mainly cortical areas, including the PFC, showed major attributions for relearning when the involvement of glutamatergic receptors (Joffe, 2019) or PFC Glx levels as predictor for reversal learning performance could be reported in marmosets (Lacreuse, 2018). Nevertheless, the absence of Glx changes after 3 weeks of associative relearning is in line with previous MRS studies pointing to acute adaptions in glutamate levels during learning (Stanley, 2017; Thielen, 2018), but failing to report long-lasting changes. Although, the hippocampus exhibits an outstanding role for memory formation in the human brain, no independent relearning-induced changes of neurotransmitter levels could be shown.

However, we could report a significant interaction of Glx/tCr levels of the hippocampus and thalamus after SSRI treatment. The thalamus serves as a mediator in top-down regulation of negative emotions by interconnecting a variety of areas associated with MDD (Price and Drevets, 2010) such as serving as an integral part of the subcortical mood circuit, connecting the prefrontal cortex with the hippocampus and amygdala (Kafetzopoulos, 2018). A pharmacological interplay of hippocampal and thalamic glutamatergic action has been previously reported when a study by (Zhang, 2012) showed imposed gamma oscillations in the hippocampus following the actions of the NMDAR antagonist ketamine in the thalamus. Several PET studies concluded that the thalamus is involved in serotonergic action and therefore a potential target for antidepressant drugs (van Harten, 1993 Smith, 1999). Although the thalamus is rich in serotonin transporter (Kish, 2005), it shows lower levels of serotonergic receptors compared to the hippocampus (Varnas, 2004). Hence, reduced serotonin receptor densities on glu-tamatergic cells in the thalamus compared to the hippocampus may explain opposed effects of SSRI treatment on Glx/tCr ratios. While, thalamic Glx/tCr levels showed no significant changes after SSRI treatment compared to placebo when each ROI was investigated individually, we could report alterations of hippocampal Glx/tCr levels. Due to the variety of serotonergic receptors on GABAergic and glutamatergic neurons in the hippocampus, a cross-talk between neurotransmitter systems is standing to reason (Dale, 2016). Hence, it is not surprising that modulators of the serotonergic system, e.g. SSRIs, induce alterations of cognitive abilities in depressive patients (Herrera-Guzman, 2010; McIntyre, 2013; McIntyre, 2014), whereby an indirect modulation of the glutamatergic and GABAergic system, that promotes neuroplastic processes, can be assumed. Underlying neurobiological actions of SSRI treatment leading to Glx/tCr decrease in the hippocampus remain a subject of interest. It was previously shown that serotonergic agents interact with gluta-matergic neurotransmission and show tendencies to down-regulate its function via serotonergic heteroreceptors on glutamatergic neurons reducing glutamate release (Pittaluga, 2007). A study conducted in rats demonstrated impaired LTP expression at a postsynaptic level after esc-italopram treatment, which supports the role of allosteric binding of the serotonin transporter in the regulation of long-lasting synaptic plasticity and neuronal firing rate (Mnie-Filali, 2006). However, modifications of the glutamate-glutamine cycle are not solely attributed to neuronal populations via the glutamate release but also show significant effects on glia cells, since serotonin receptors are found both on neurons and astrocyte populations (Jakab and Goldman-Rakic, 1998, 2000). Synaptic glutamate levels may also be directly affected by glial-specific glutamate transporters, whose expression levels showed alterations after antidepressant treatment (Czeh and Di Benedetto, 2013). Moreover, chronic treatments of rats with SSRIs, norepinephrine reuptake inhibitors or tricyclic antidepressants lead to reduced depolarization-evoked glutamate release in the hippocampus (Bonanno, 2005). Thus, it can be speculated, that SSRI-induced increased serotonin levels in the human hippocampus lead to reduced glutamate release, as found in rats, which is reflected in decreased Glx/tCr levels.

Previous MRS studies investigating effects of increased serotonin levels after SSRI treatment on GABAergic and glutamatergic neurotransmission mainly reported changes of GABA. The cingulate and occipital cortex showed adaptions of total GABA levels after acute (Bhagwagar, 2004; Brennan, 2017) or long-lasting treatment with SSRIs (Sanacora, 2002). However, in this study, no effects of relearning and treatment on GABA+/tCr ratios could be shown in any of the ROIs investigated, which may point to the conclusion that GABAergic adaptions are restricted to higher order control areas of the limbic system rather than subcortical structures. Similar to GABA findings, alterations in the glu-tamatergic system after SSRI treatment investigated with MRS showed a regionally diverse pattern since Taylor and colleagues reported no effects of citalopram treatment in healthy subjects after one week of treatment in the frontal cortex (Taylor, 2010), while an increase in Glx in the occipital cortex was found (Taylor, 2008). Nevertheless, the reported decrease of Glx/tCr in the hippocampus after SSRI treatment compared to placebo provides valuable insights in the action of SSRI treatment, especially in MDD. Patients with MDD tend to show reduced Glx levels in the hippocampus among other areas compared to healthy controls (Michael, 2003; Yildiz-Yesiloglu and Ankerst, 2006; Block, 2009). However, previous studies did not find increased Glx levels after SSRI treatment in the hippocampus in patients but, to the contrary, similar to our study, a statistical trendwise reduction of Glx (Block, 2009). Decreased hippocampal glutamate levels may counteract overreactivity in the frontolimbic circuitry, which was shown in fMRI studies of patients with depression (Harvey, 2005; Walsh, 2007).

### 4.1 Limitations

Citalopram plasma levels were assessed for three times during the administration period of 21 days to control for general compliance of the study subjects regarding oral intake of the study drug. However, we ultimately cannot exclude that some subjects did not take the study drug on a daily basis. It cannot be excluded that the applied relearning stimulus of 15 min relearning and retrieval per day was too short, and therefore too weak to independently evoke changes in neurotransmitter levels. Moreover, we were unable to control for learning of participants beyond the study scope. We performed stringent quality control (CRLB thresholds) on MRS data and while this results in missing values, imputation allows for application of the selected statistical procedures. Nevertheless, averaging of imputed values as performed reduces variance. Moreover, the rank transform might have resulted in reduced sensitivity and led to false negative results of GABA-related measures. Furthermore, it has to be considered that MRS does not allow distinguishing intra- and extracellular neurotransmitter levels. While the use of MRSI yields several advantages, signal spill-over from adjacent voxels cannot be excluded. Thus, spill-over effects can be reduced using higher spatial resolution at higher field strengths (Moser, 2019).

### 4.2 Conclusion

Here, we investigated the effects of associative relearning and SSRI treatment on brain GABA+/tCr and Glx/tCr ratios in a placebo-controlled double-blind design. The absence of relearning induced differences of neurotransmitter levels with or without emotional connotation may be explained by acute and not long-lasting neurotransmitter adaptions during associative learning (Stanley, 2017; Thielen, 2018). However, the revealed hippocampus-thalamus interaction effect of Glx/tCr ratios, potentially arising from higher serotonin transporter and lower serotonin receptor distributions in the thalamus, highlights the importance of using MRSI in clinical studies. Moreover, we showed hippocampal changes of Glx/tCr levels after SSRI treatment. These changes reflect the broad spectrum of possible adaptations of the glutamatergic system, a system that is not the primary target of SSRIs. Nevertheless, underlying neurobiological actions remain challenging to investigate, since regulation of hippocampal function by serotonin is complex, involving multiple receptor subtypes expressed on glutamatergic cells and GABAergic interneurons. Nevertheless, SSRI-induced reductions of glutamate release can be speculated. Future studies should aim to include the PFC, a region highly connected to the hippocampus and thalamus and an essential component for both learning and psychiatric disease, and may shift to higher field strengths allowing the separation of glutamate and glutamine using MRS (Gruber, 2017) in health and patients with MDD.

## Supplementary Material

Supplementary material associated with this article can be found, in the online version, at doi:10.1016/j.neuroimage.2021.117913.

Supplementary

## Figures and Tables

**Table 1:** Number of imputations for each ROI and metabolite.

		hippocampus	insula	putamen	pallidum	thalamus
GABA+/tCr	M1	13 (22.4%)	7 (12.1%)	3 (5.2%)	3 (5.2%)	1 (1.7%)
	M2	7 (12.1%)	2 (3.4%)	3 (5.2%)	2 (3.4%)	0 (0%)
Glx/tCr	M1	1 (1.5%)	3 (4.5%)	2 (3.0%)	1 (1.5%)	1 (1.5%)
	M2	3 (4.5%)	0 (0%)	3 (4.5%)	2 (3.0%)	0 (0%)

**Table 2:** Detailed group size for each analysis.

					char. relearning		facial relearning	
	SSRI(total)	placebo(total)	facial relearning(total)	char. relearning(total)	placebo	SSRI	placebo	SSRI
GABA+/tCr	21	37	27	31	20	11	17	10
Glx/tCr	29	37	30	36	20	16	17	13

**Table 3:** Mean ratios and standard deviation for each ROI within each group and measurement.

		group 1		group 2		group 3		group 4	
		character/placebo		character/SSRI		facial/placebo		facial/SSRI	
		M1	M2	M1	M2	M1	M2	M1	M2
**hippocampus**	**GABA+/tCr**	0.24±0.02	0.25±0.03	0.24±0.03	0.25±0.04	0.26±0.03	0.27±0.02	0.24±0.03	0.24±0.04
	**Glx/tCr**	1.42±0.21	1.51±0.28	1.58±0.40	1.49±0.37	1.50±0.12	1.60±0.20	1.73±0.64	1.47±0.22
**insula**	**GABA+/tCr**	0.25±0.03	0.26±0.03	0.25±0.02	0.27±0.04	0.26±0.03	0.26±0.04	0.26±0.04	0.26±0.05
	**Glx/tCr**	1.55±0.12	1.60±0.21	1.71±0.29	1.58±0.21	1.66±0.26	1.65±0.14	1.67±0.32	1.54±0.14
**putamen**	**GABA+/tCr**	0.28±0.04	0.30±0.05	0.29±0.03	0.30±0.04	0.29±0.05	0.29±0.04	0.30±0.05	0.28±0.04
	**Glx/tCr**	1.52±0.17	1.58±0.26	1.66±0.31	1.60±0.26	1.65±0.21	1.61±0.15	1.56±0.19	1.49±0.21
**pallidum**	**GABA+/tCr**	0.29±0.04	0.31±0.05	0.31±0.03	0.31±0.04	0.31±0.05	0.33±0.03	0.30±0.04	0.28±0.04
	**Glx/tCr**	1.51±0.24	1.57±0.27	1.56±0.33	1.54±0.35	1.54±0.18	1.57±0.23	1.44±0.24	1.51±0.59
**thalamus**	**GABA+/tCr**	0.31±0.05	0.31±0.04	0.31±0.04	0.32±0.04	0.32±0.04	0.33±0.03	0.31±0.04	0.31±0.05
	**Glx/tCr**	1.38±0.18	1.42±0.21	1.46±0.22	1.52±0.24	1.45±0.21	1.48±0.17	1.33±0.19	1.56±0.59

## Data Availability

Clinical datasets of participants presented in this article are not readily available due to ethical reasons. Please contact rupert.lanzenberger@meduniwien.ac.at for questions.
