# Supplementary material for: Effects of SSRI treatment on GABA and glutamate levels in an associative relearning paradigm
Source: Neuroimage. Author manuscript; Available in PMC 2022 May 15. (PMC7610796; doi:10.1016/j.neuroimage.2021.117913)
Supplement: Supplementary [file EMS123951-supplement-Supplementary.docx]

**Supplement material:**


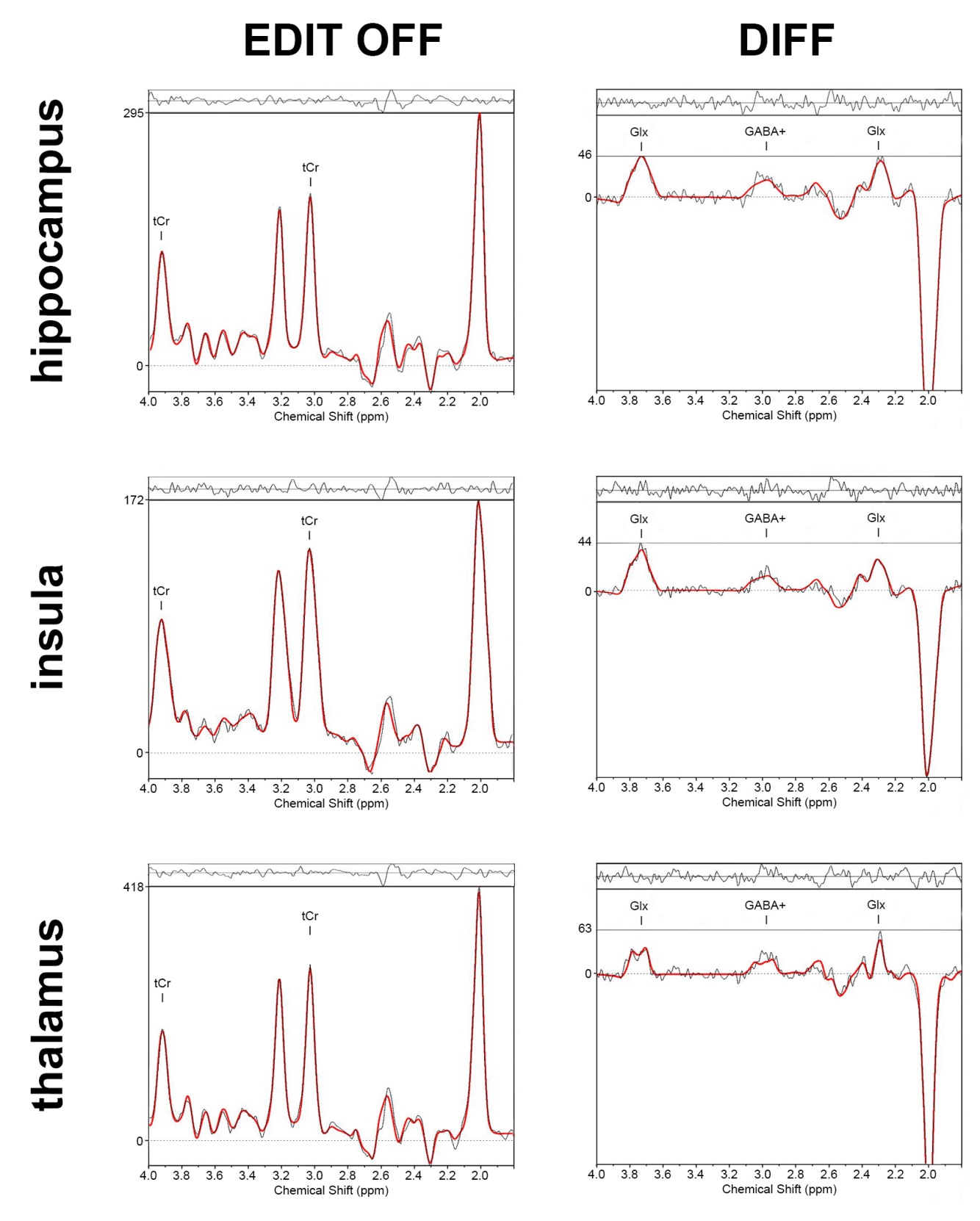


**Supplement figure 1:** Exemplary spectra from the hippocampus, insula and putamen. Exemplary LCModel fits of the edit off and difference spectra for the hippocampus, insula and thalamus. GABA+ (GABA and macromolecules), glutamate and glutamine (Glx) and total creatine (tCr) peaks are marked.

**Supplement table 1**: Mean ratios and standard deviation for each ROI within each relearning and treatment group

|  |  | **characters** | | **faces** | | **SSRI** | | **placebo** | |
| --- | --- | --- | --- | --- | --- | --- | --- | --- | --- |
|  |  | **M1** | **M2** | **M1** | **M2** | **M1** | **M2** | **M1** | **M2** |
| **hippocampus** | **GABA+/tCr** | 0.24±0.02 | 0.25±0.03 | 0.25±0.03 | 0.26±0.03 | 0.24±0.03 | 0.25±0.04 | 0.25±0.03 | 0.26±0.03 |
|  | **Glx/tCr** | 1.49±0.31 | 1.50±0.32 | 1.60±0.44 | 1.55±0.21 | 1.65±0.52 | 1.48±0.30 | 1.45±0.18 | 1.55±0.25 |
| **insula** | **GABA+/tCr** | 0.25±0.03 | 0.26±0.03 | 0.26±0.04 | 0.26±0.04 | 0.26±0.03 | 0.27±0.04 | 0.26±0.03 | 0.26±0.03 |
|  | **Glx/tCr** | 1.62±0.23 | 1.59±0.21 | 1.67±0.28 | 1.60±0.15 | 1.70±0.30 | 1.56±0.18 | 1.60±0.20 | 1.62±0.18 |
| **putamen** | **GABA+/tCr** | 0.28±0.04 | 0.30±0.04 | 0.30±0.05 | 0.29±0.04 | 0.30±0.04 | 0.29±0.04 | 0.28±0.05 | 0.29±0.04 |
|  | **Glx/tCr** | 1.58±0.25 | 1.59±0.25 | 1.61±0.20 | 1.56±0.18 | 1.62±0.27 | 1.55±0.24 | 1.58±0.20 | 1.60±0.21 |
| **pallidum** | **GABA+/tCr** | 0.30±0.04 | 0.31±0.05 | 0.31±0.05 | 0.31±0.04 | 0.31±0.04 | 0.30±0.05 | 0.30±0.05 | 0.32±0.04 |
|  | **Glx/tCr** | 1.53±0.28 | 1.56±0.30 | 1.50±0.21 | 1.54±0.42 | 1.51±0.30 | 1.53±0.47 | 1.52±0.21 | 1.57±0.25 |
| **thalamus** | **GABA+/tCr** | 0.31±0.05 | 0.32±0.04 | 0.31±0.04 | 0.32±0.04 | 0.31±0.04 | 0.31±0.05 | 0.31±0.05 | 0.32±0.04 |
|  | **Glx/tCr** | 1.41±0.20 | 1.47±0.23 | 1.40±0.21 | 1.52±0.40 | 1.40±0.21 | 1.54±0.43 | 1.41±0.19 | 1.45±0.19 |
